# Prevalence, risk factors, and clinical impact of restless leg syndrome on uremia patients with hemodialysis

**DOI:** 10.3389/fneur.2026.1929976

**Published:** 2026-09-09

**Authors:** Yuan Xu, Jie Li, Lianchen Xiao, Kai Luo, Jing Yang, Hongbin Wen

**Affiliations:** 1 Department of Neurology, Xiangyang Central Hospital, Affiliated Hospital of Hubei University of Arts and Science, Xiangyang, China; 2 Comprehensive Stroke Center, Xiangyang Central Hospital, Affiliated Hospital of Hubei University of Arts and Science, Xiangyang, China; 3 School of Food Science and Chemical Engineering, Hubei University of Arts and Science, Xiangyang, China

**Keywords:** hemodialysis, life quality, restless legs syndrome, sleep quality, uremia

## Abstract

**Background:**

Restless Legs Syndrome (RLS) seriously affects the sleep and life quality of patients, yet it often leads to delay in diagnosis and treatment due to a lack of awareness. Clinical studies on RLS, particularly regarding its epidemiology in uremia patients, remain relatively scarce.

**Objective:**

This study conducted a clinical analysis to assess the frequency of RLS among hemodialysis patients and to explore its impact on their sleep and quality of life.

**Methods:**

We collected clinical data and laboratory results from 147 uremia patients undergoing maintenance hemodialysis at Xiangyang Central Hospital. Independent risk factors for RLS were evaluated using univariate and multivariate logistic regression. Sleep quality and quality of life were assessed using the Pittsburgh Sleep Quality Index (PSQI) and the 36-item Short-Form Health Survey (SF-36), respectively. We compared these outcomes between the RLS and non-RLS groups.

**Results:**

Among the 147 patients, 30 (20.41, 95% CI, 14.0–27.0%) were diagnosed with RLS. Multivariate logistic regression analysis, performed with caution due to the limited number of RLS cases (*n* = 30), identified β2-microglobulin, dialysis duration, and parathyroid hormone as potential independent risk factors for RLS in this population. Compared to the non-RLS group, patients with RLS had significantly lower physiological function scores on the SF-36 (*p* < 0.05). Furthermore, the RLS group exhibited significantly higher PSQI scores in the domains of subjective sleep quality, sleep persistence, habitual sleep efficiency, and daytime dysfunction (*p* < 0.05).

**Conclusion:**

The incidence of RLS is notably elevated in uremia patients on maintenance hemodialysis. RLS significantly impairs both sleep quality and the physiological function of these patients. Early identification and management of RLS are crucial for improving their overall well-being. Further large-scale studies are warranted to confirm the identified risk factors.

**Clinical trial registration:**

MedicalResearch.org.cn, MR-42-24-017773.

## 1 Introduction

Restless Legs Syndrome (RLS) is a sensorimotor disorder characterized by unpleasant leg sensations and an irresistible urge to move, typically worsening during rest or at night (1, 2), which is frequently misdiagnosed (3, 4). The syndrome can occur at any age but is more prevalent among the elderly (5–7). RLS can be primary (8) or secondary to conditions such as uremia (9–11), was first comprehensively described by Ekbom (12). Population studies estimate the prevalence of RLS to be between 5 and 15% (13, 14). The syndrome can severely diminish daytime productivity and quality of life. However, current diagnostic approaches often lack accuracy and timeliness, and the pathophysiology remains incompletely understood. Consequently, RLS is receiving increasing global attention.

Although RLS significantly impairs sleep and quality of life, it remains underrecognized, and data on its epidemiology in uremia patients undergoing hemodialysis are scarce in China (15–17). As a country with a large population, China has more than two million uremia patients (18), while the health and life quality of the uremia patients have attracted more and more attention. Studies shown that the incidence of RLS in hemodialysis patients with uremia could be as high as 20–57%, which deteriorates the patient’s quality of life and in severe cases it could lead to increased mortality (19–21).

Although advancements in hemodialysis technology have improved the life expectancy of patients with end-stage renal disease (ESRD), these individuals remain susceptible to various complications, furthermore, data on the incidence of RLS among uremic patients are limited. Studies suggested that ESRD defined as irreversible, advanced kidney failure requiring renal replacement therapy, appeared to be a risk factor for the development or exacerbation of RLS (22–24). Iron deficiency, common in uremia, may contribute to RLS through potential mechanisms such as anemia and altered dopamine metabolism in the central nervous system (25). Given its chronic nature and significant impact on the health and quality of life of uremic patients, RLS warrants greater clinical attention. However, the pathophysiology of RLS in uremia remains unclear. Limited research focus and insufficient awareness among clinicians and patients often lead to missed diagnoses and misdiagnosis of RLS in this population (14, 26). RLS-induced sleep disturbances result in insufficient rest, daytime fatigue, and impaired attention. Compared to healthy individuals, affected patients experience higher rates of anxiety, depression, chronic pain, and cognitive impairments, severely compromising their quality of life (27). Moreover, worsening RLS severity correlates with aggravated depressive symptoms and further reductions in both sleep and overall quality of life (28). Consequently, RLS represents a significant neurological comorbidity in uremic patients, making its prevention, timely diagnosis, and effective management of considerable clinical importance.

In this study, the prevalence of RLS was investigated, and uremia patients were analyzed to explore the incidence factors and influence of RLS in uremia patients on hemodialysis, to help the diagnosis and treatment of RLS and improve the quality of life in uremia patients.

## 2 Methods

### 2.1 Study design and participants

A total of 147 uremia patients on hemodialysis admitted to the Xiangyang Central Hospital in Xiangyang City from January 2024 to January 2025 were selected as the research subjects. The study was approved by the Ethics Committees of Xiangyang Central Hospital, the Affiliated Hospital of Hubei University of Arts and Science, Xiangyang, China. All participants were informed about the investigative nature of the study, the use of their personal data and the signed informed consent form before any study- related procedure.

### 2.2 Inclusion and exclusion criteria

Patients were included if they met the following criteria: (1) aged 18 years or older; (2) had undergone hemodialysis treatment for more than 3 months; (3) had no change in dialysis mode or surgical history within 3 months before enrollment; and (4) had been clinically stable for at least one month.

Patients were excluded if they presented with any of the following: (1) severe respiratory, cardiovascular, hematological, or hepatic diseases; (2) insomnia caused by systemic diseases (e.g., pain, surgery) or external environmental interference; (3) personality disorders, communication disorders, intellectual or thinking abnormalities, or mental abnormalities.

### 2.3 Research measures

Clinical data of hemodialysis patients were collected, including patient gender, age, age of dialysis, chosen dialyser membrane materials, and laboratory indexes including serum creatinine, blood urea, red blood cells deposited, hemoglobin, serum ferritin, calcium, phosphorus, transferrin saturation, β2- microglobulin and parathyroid hormone. A cross-sectional control study was used. All the subjects were surveyed and tested in the form of face-to-face interview. To minimize bias, investigators were trained to conduct interviews uniformly, and key findings were verified with family members where possible.

Hemodialysis methods: all 147 hemodialysis patients were treated with Fresenius 4008S (Fresenius, Germany) dialysis machine. The dialysate used the bicarbonate solution with a flow rate of 500 mL/min, and dialyzer used the cellulose triacetate membrane or polysulfone membrane. The dialysis time was 4 h each time, and the blood flow was controlled within 200–300 mL/min. All assessments, including RLS diagnosis and questionnaire administration, were conducted on a mid-week, non-dialysis day to avoid the immediate effects of the dialysis session.

RLS severity was assessed using the International Restless Legs Syndrome Study Group Rating Scale (IRLS). The IRLS consists of 10 items, each rated on a 0–4 scale, with a total score ranging from 0 to 40. Severity was classified as follows: mild (1–10), moderate (11–20), severe (21–30), and very severe (31–40). The diagnosis of RLS was established by trained neurologists based on the 2014 revised diagnostic criteria of the International Restless Legs Syndrome Study Group (IRLSSG) (29), which requires all five essential criteria to be met.

The Pittsburgh Sleep Quality Index (PSQI) was utilized to assess patients’ sleep quality over the preceding month (30, 31). This questionnaire comprises 23 items grouped into seven components: subjective sleep quality, sleep latency, sleep duration, habitual sleep efficiency, sleep disturbances, use of sleep medication, and daytime dysfunction (32). The global PSQI score ranges from 0 to 21, with higher scores indicating poorer sleep quality. A score exceeding 7 is indicative of poor sleep quality. Both global and component scores were analyzed.

The 36- item shot- form health status survey (SF- 36) was used to observe the life quality of patients (33, 34). SF- 36 evaluates the 8 aspects of life quality, which were physical function (PF), body pain (BP), role physical (RP), general health (GH), social function (SF), role emotional (RE) and vitality (VT) and mental health (MH), respectively. In the scoring process, each item was firstly forward processed and scored item by item, and then the original score was converted to 0–100 standard score according to the SF-36 standard scoring conversion formula. The higher the score of each dimension of patients is, the better their quality of life is.

### 2.4 Quality control

(1) Training of investigators: Neurology physicians were trained by Xiangyang Central Hospital to ensure the uniformity and accuracy of the investigation. (2) Quality control of the investigation process: investigators with unified training will conduct a face- to- face investigation of RLS patients and verify the results of sleep and mood investigation with family members to ensure the authenticity and accuracy of the investigation. (3) Diagnostic criteria for RLS: IRLSSG revised the diagnostic criteria for RLS in 2014 (29), and excluded PLMS, vascular diseases of lower limbs and other diseases that need to be differentiated. (4) PSQI was used to evaluate the sleep status of the patients in the past month, and the subjective sleep quality, sleeping time, sleep duration, sleep efficiency, sleep disorder, use of hypnotic drugs and daytime dysfunction were asked item by item, and the scores were used to determine whether the patients had sleep disorders.

### 2.5 Statistical analysis

Continuous variables were tested for normality using the Shapiro–Wilk test. For variables that did not follow a normal distribution, comparisons between groups were performed using the Mann–Whitney U test. Normally distributed variables were compared using the independent samples *t*-test. Categorical variables were compared using the Chi-square test or Fisher’s exact test, as appropriate. Univariate and multivariate logistic regression analyses were used to identify risk factors for RLS. Given the limited number of RLS cases (*n* = 30), the multivariate model was restricted to three pre-selected variables (β2-microglobulin, dialysis duration, and parathyroid hormone) based on clinical relevance and univariate significance to minimize the risk of model overfitting. The events-per-variable ratio was approximately 10:1, which is more acceptable than the initial 6.7:1 ratio that would have resulted from including all univariate-significant variables. All analyses were performed using SPSS software (version 22.0). Statistical significance was defined as a two-tailed *p* < 0.05. Data are presented as the mean of quadruplicate measurements ± standard deviation (SD).

## 3 Results

### 3.1 Demographic characteristics of the study population

Among the 147 enrolled patients, 86 were male and 61 were female. The patients’ age ranged from 18 to 80 years, with a mean age of 54.39 years. The dialysis duration ranged from 3 to 104 months, with a mean of 41.28 months.

### 3.2 The incidence of RLS in hemodialysis patients

Among the 147 uremia patients with maintenance hemodialysis (MHD), 30 patients with RLS had a prevalence of 20.41% (95% CI: 14.0–27.0%). According to the severity of RLS, there were 7 patients with mild disease, 23 patients with moderate disease, and 0 patients with severe disease. The average severity score was 15.62 ± 3.97.

### 3.3 Univariate analysis of influencing the incidence of RLS

Univariate analysis was conducted for the related factors of the patients, and the results (Table 1) showed that there were statistically significant differences in gender, age, dialysis duration, weekly dialysis time, phosphorus, β2- microglobulin and parathyroid hormone (PTH) between the RLS group and the non-RLS group (*p* < 0.05).

**Table 1:** Baseline characteristics of patients with and without Restless Leg Syndrome.

Factors	RLS (*n* = 30)	Non-RLS (*n* = 117)	*t* /χ^2^	*p*
Gender (Male/female)	12/18	78/39	5.198	0.023
Age (Years)	56.38 ± 8.73	50.52 ± 10.21	2.418	0.017
DM (PS/CTA)	13/17	84/33	0.382	0.537
DD (Months)	50.67 ± 11.25	38.47 ± 7.35	6.291	0.003
IW (kg)	62.94 ± 9.85	61.88 ± 12.36	0.364	0.716
DT (hours per week)	12.39 ± 1.46	10.21 ± 2.15	4.359	0.001
Scr (μmol/L)	983.52 ± 196.47	964.58 ± 215.30	0.368	0.713
BUN (mmol/L)	27.84 ± 5.32	26.59 ± 6.45	0.820	0.412
HGB (g/L)	114.39 ± 28.65	121.37 ± 30.46	0.955	0.340
Ca (mmol/L)	2.45 ± 0.35	2.36 ± 0.44	0.868	0.386
P (mmol/L)	1.83 ± 0.68	1.41 ± 0.46	3.493	0.004
HCT (%)	34.68 ± 7.21	35.62 ± 6.12	0.618	0.633
SF (μg/mL)	337.58 ± 80.46	350.83 ± 74.39	0.728	0.431
TSAT (%)	25.69 ± 3.30	26.83 ± 4.25	1.141	0.254
β2-MG (μg/L)	34.20 ± 10.95	13.47 ± 5.22	13.498	0.002
PTH (ng/L)	483.68 ± 120.58	186.92 ± 48.73	19.185	0.001

DM, dialysis membrane; PS, polysulfone; CTA, cellulose triacetate; DD, dialysis duration; IW, individual weight; DT, dialysis time; Scr, serum creatinine; BUN, blood urea nitrogen; HGB, hemoglobin; Ca, calcium; P, phosphorus; HCT, hematokrit; SF, serum ferritin; TSAT, transferrin saturation; β2-MG, β2-microglobulin; PTH, parathyroid hormone.

Each value represents the mean of four replicates ± standard deviation, hereinafter inclusive.

### 3.4 Logistic multivariate regression analysis of influencing the incidence of RLS

The factors with statistical significance in the univariate analysis were analyzed by Logistic multivariate regression analysis. To avoid model overfitting due to the limited number of RLS events (*n* = 30), we restricted the number of variables in the model. Based on clinical relevance and the strength of their univariate associations, β2-microglobulin, dialysis duration, and PTH were entered into the regression model. The analysis identified these three factors as independent risk factors affecting the occurrence of RLS in uremia hemodialysis patients (*p* < 0.05), as shown in Table 2.

**Table 2:** Logistic multivariate regression analysis of influencing the incidence of RLS.

Factors	PRC	SE	Wald χ^2^	*p*	OR	OR 95% CI	
						Min	Max
β2-MG	2.835	0.722	16.915	0.000	3.268	6.736	16.268
DD	2.197	0.573	12.678	0.021	2.557	4.672	13.578
PTH	1.975	0.352	10.683	0.034	1.863	3.732	12.744

PRC, partial regression coefficent; SE, standard error; OR, odds ratio; CI, confidence interval; β2-MG, β2-microglobulin; DD, dialysis duration; PTH, parathyroid hormone.

### 3.5 Influence of RLS patients on quality of life

The physiological function score of SF- 36 in RLS group was significantly lower than that in non- RLS group (*p* < 0.05), while there was no significant difference in other dimensions between the two groups (*p* > 0.05), as shown in Table 3.

**Table 3:** Impact scores for RLS and non-RLS patients.

Dimension	RLS (*n* = 30)	Non-RLS (*n* = 117)	*t*	*p*
PF	58.67 ± 12.59	79.53 ± 10.21	10.510	0.008
BP	75.28 ± 10.25	78.35 ± 12.33	1.384	0.166
RP	48.52 ± 9.48	51.37 ± 8.65	1.744	0.081
GH	44.39 ± 9.51	46.83 ± 12.30	1.113	0.26
SF	68.56 ± 13.17	70.45 ± 12.26	0.819	0.412
RE	70.55 ± 11.64	72.84 ± 9.76	1.217	0.225
VT	62.10 ± 7.85	65.44 ± 15.04	1.289	0.197
MH	57.49 ± 13.47	59.06 ± 10.58	0.760	0.447

PF, physical function; BP, body pain; RP, role physical; GH, general health; SF, social function; RE, role emotional; VT, vitality; MH, mental health.

### 3.6 Influence of RLS patients on sleep quality

**Figure 1:**
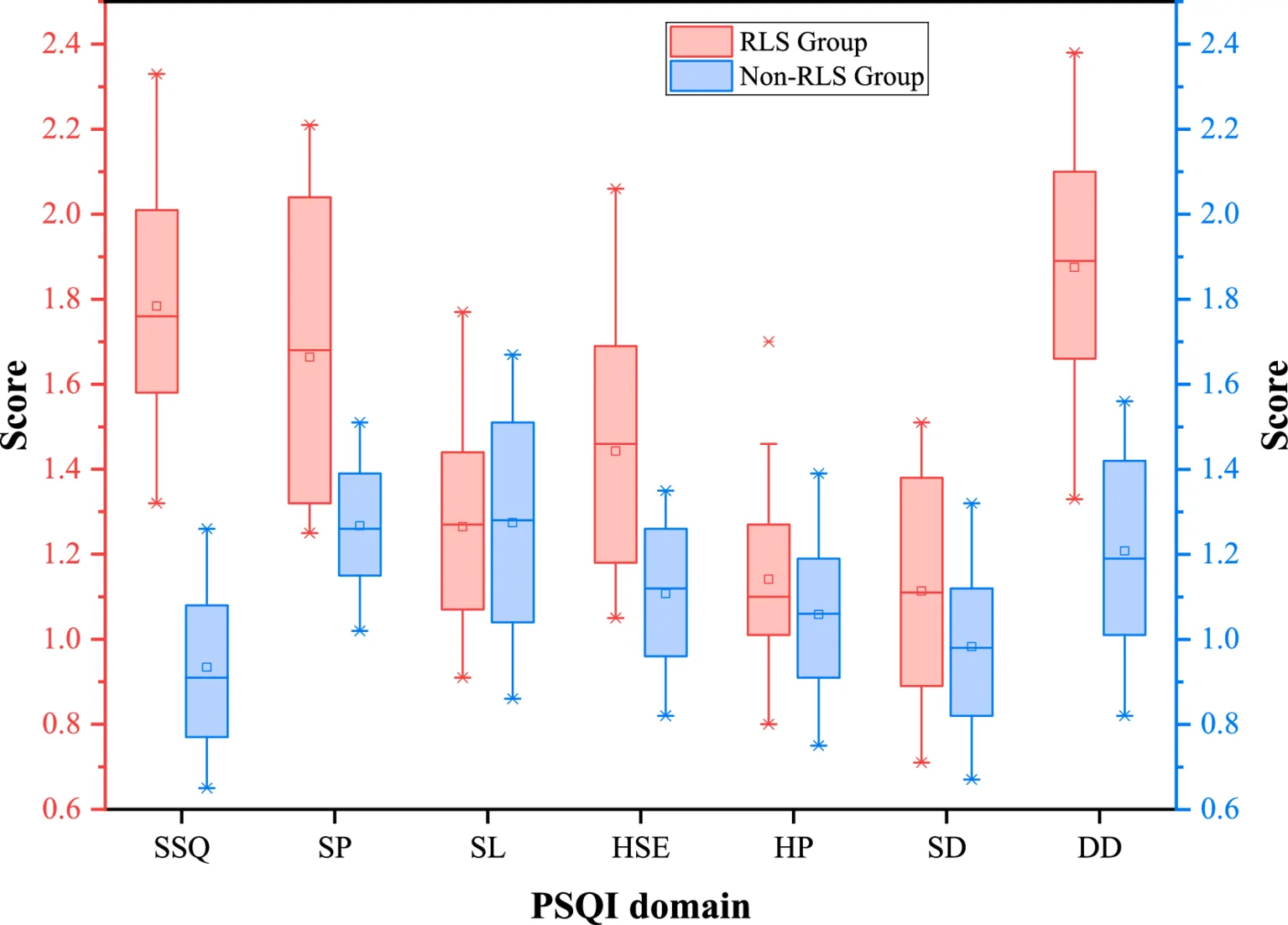
Box chart of factors affecting sleep quality in two groups of patients.

Subjective sleep quality, sleep persistence, habitual sleep efficiency and daytime dysfunction PSQI score in RLS group were significantly higher than those in non-RLS group (*p* < 0.05) (Table 4). The average of daytime dysfunction (1.86) and sleep disturbance (1.11) were the highest and lowest in the RLS group, while non-RLS group had the highest mean sleep latency (1.27) and the lowest mean subjective sleep quality (0.95) (Figure 1).

**Table 4:** Influence score of RLS on sleep quality of patients.

Factors	RLS (*n* = 30)	Non-RLS (*n* = 117)	*t*	*p*
SSQ	1.83 ± 0.51	0.95 ± 0.30	13.574	0.005
SP	1.73 ± 0.48	1.25 ± 0.33	7.126	0.001
SL	1.34 ± 0.43	1.27 ± 0.41	0.912	0.362
HSE	1.46 ± 0.41	1.08 ± 0.27	6.795	0.001
HP	1.12 ± 0.35	1.07 ± 0.32	0.827	0.408
SD	1.11 ± 0.40	0.99 ± 0.33	1.879	0.060
DD	1.86 ± 0.53	1.18 ± 0.37	9.049	0.001

SSQ, Subjective sleep quality; SP, Sleep persistence; SL, Sleep latency; HSE, habitual sleep efficiency; HP, hypnotic; SD, sleep disorder; DD, daytime dysfunction.

## 4 Discussion

RLS can occur at any age, with a higher incidence in women than in men. Some investigations shown that RLS was more prevalent in uremic females (22, 35). RLS occurs at a lower frequency among hemodialysis patients but is a major problem for them (36, 37). However, in patients with end-stage renal disease (ESRD) undergoing hemodialysis, the prevalence is substantially higher, generally reported between 10 and 20% (38), and some studies have reported rates as high as 20–57% (39). In the present study, among 147 uremia patients on maintenance hemodialysis, 30 patients (20.41%) were diagnosed with RLS, which is consistent with the 10–20% range reported in the nephrology literature (38). Notably, this prevalence is comparable to the 14.5% reported by Xiao et al. in a multicenter survey of 269 maintenance hemodialysis patients (40), and falls within the 18.6% frequency reported by Kawacchi et al. among chronic hemodialysis patients (39). RLS was an important source of sleep disruption, and medications was important predictors for RLS (41). The incidence of RLS increases with age and reaches its peak at the age of 45–54. Dong et al. (42) retrospective analysis of the PubMed database and comprehensive epidemiological analysis of the prevalence of RLS found that the prevalence of RLS in the general population was between 5 to 15%. In this study, among 147 hemodialysis patients in our hospital, 30 patients suffered from RLS, and the prevalence rate of RLS patients was 20.41%. There was a significant correlation between RLS and physiological functions of SF- 36, which was similar to the results of Akba and other studies (43, 44). These findings suggest that the prevalence of RLS in Chinese hemodialysis patients is not substantially different from that in other hemodialysis populations, though it remains higher than that of the general population.

The pathogenesis and mechanism of RLS in uremia patients are not clear at present (45). Some studies suggested that it might be due to poor compliance with therapy, impaired health-related quality of life, depression, and the psychological consequences of recurrent sleep disruption in these patients (46–48). The presence of RLS is associated to longer durations of dialysis (49). Some studies showed that high- efficiency online hemodiafiltration was an effective therapeutic strategy for restless legs syndrome in dialysis patients (50–52). It was reported that iron- deficiency anemia, elevated serum calcium, central nervous system abnormalities, and peripheral neuropathy secondary to uremia may predispose uremia patients to developing RLS (25, 53, 54). This study analyzed the uremia patients on hemodialysis, and the results showed that the β2- microsphere, dialysis duration and PTH were independent risk factors affecting the occurrence of RLS in uremia patients on hemodialysis. It showed that the incidence of RLS was closely related to the renal function and dialysis time of patients. However, these findings require validation in larger, independent cohorts. The limited number of RLS events in our study (*n* = 30) means that the multivariate model may be susceptible to overfitting, and the odds ratios should be interpreted with caution. Unmeasured confounders, such as medication use, diabetes, and peripheral neuropathy, could also influence the observed associations and were not accounted for in our analysis.

This study also compared the quality of life and sleep quality between hemodialysis patients with and without RLS. The results showed that RLS patients had significantly higher PSQI scores in subjective sleep quality, sleep persistence, habitual sleep efficiency, and daytime dysfunction, indicating that RLS adversely affects multiple dimensions of sleep in uremia patients on hemodialysis. These findings are consistent with those of Xiao et al., who reported that RLS patients had significantly higher PSQI scores (*p* < 0.001), reduced subjective sleep quality (*p* < 0.001), increased sleep latency (*p* = 0.007), shorter sleep duration (*p* < 0.001), lower sleep efficiency (*p* = 0.001), higher sleep disturbances (*p* < 0.001), and increased daytime dysfunction (*p* = 0.019) (40). In terms of quality of life, the SF-36 physiological function score in the RLS group was significantly lower than that in the non-RLS group (*p* < 0.05), while no significant differences were observed in other dimensions. Kawacchi et al. similarly found that RLS leads to disturbances in physical quality of life (39). However, researchers emphasized that RLS in dialysis patients is associated with not only physical but also emotional symptoms that directly affect overall quality of life and healthcare utilization (38). The relatively limited impact on other SF-36 dimensions in our study may be attributed to the predominance of mild-to-moderate RLS cases (no severe cases were identified) and the relatively small sample size. It is also important to note that the cross-sectional design precludes establishing causality between RLS and the observed sleep and quality of life impairments; a bidirectional relationship is possible.

The study has several limitations. Firstly, this research was conducted in a single hospital. Patients with uremia undergoing dialysis in other hospitals may have different conditions such as severity of illness and living environment. Comparative studies will be carried out in other hospitals in the future. Secondly, a total of 147 patients were included in the study, the relatively limited sample size, particularly with only 30 RLS cases, constrains the statistical power for multivariate modeling and increases the risk of overfitting. Thirdly, this is a cross-sectional study, which precludes establishing causal relationships. In the future, longitudinal studies will be considered to further investigate the impact of restless legs syndrome on sleep and quality of life. Fourthly, the multivariate logistic regression model included only 30 RLS cases. With three independent variables entered into the model, the events-per-variable ratio was 10:1, which is acceptable but still relatively low. Model overfitting cannot be entirely ruled out, and therefore, these findings should be interpreted with caution, and larger-scale studies are warranted to validate the identified risk factors. Fifthly, we did not collect detailed information on medication use, including dopaminergic agents, alpha2-delta ligands, or iron supplementation. This is an important limitation because these medications can influence the symptoms of RLS, as well as key outcomes measured in our study. For example, dopaminergic agents and alpha2-delta ligands may affect sleep quality and quality-of-life scores, while iron supplementation could alter peripheral blood iron metabolism indicators, such as serum ferritin and transferrin saturation, potentially confounding the observed associations. Future studies should systematically document medication histories and incorporate them into the analytic framework to better account for these potential confounding effects. Finally, the reliance on subjective measures like PSQI and SF-36 introduces the potential for recall and social desirability bias.

## 5 Conclusion

The incidence of RLS is elevated in uremia patients on maintenance hemodialysis, with a prevalence of 20.41% in this study. β2-microglobulin, dialysis age, and PTH were identified as potential independent risk factors, but these findings require confirmation in larger studies. RLS significantly impairs sleep quality, particularly subjective sleep quality, sleep persistence, habitual sleep efficiency, and daytime dysfunction, and adversely affects the physiological function of these patients. Early identification and management of RLS are essential for improving the overall well-being of uremia patients on hemodialysis.

## Data Availability

The original contributions presented in the study are included in the article/supplementary material, further inquiries can be directed to the corresponding authors.
